# A cross-sectional analysis of factors associated with detection of oncogenic human papillomavirus in human immunodeficiency virus-infected and uninfected Kenyan women

**DOI:** 10.1186/s12879-019-3982-7

**Published:** 2019-04-27

**Authors:** A. Ermel, P. Tonui, M. Titus, Y. Tong, N. Wong, J. Ong’echa, K. Muthoka, S. Kiptoo, A. Moormann, J. Hogan, A. Mwangi, S. Cu-Uvin, P. J. Loehrer, O. Orang’o, D. Brown

**Affiliations:** 1 Indiana University School of Medicine, Indianapolis, IN USA; 2 Moi University, Eldoret, Kenya; 3 Maseno University, Kisumu, Kenya; 4 Kenya Medical Research Institute, Nairobi, Kenya; 5 University of Massachusetts Medical School, Worcester, MA USA; 6 Brown University, Providence, RI USA

**Keywords:** Oncogenic HPV, HIV infection, Kenyan women

## Abstract

**Background:**

Cervical cancer is caused by oncogenic human papillomaviruses (HPV) and is one of the most common malignancies in women living in sub-Saharan Africa. Women infected with the human immunodeficiency virus (HIV) have a higher incidence of cervical cancer, but the full impact on HPV detection is not well understood, and associations of biological and behavioral factors with oncogenic HPV detection have not been fully examined. Therefore, a study was initiated to investigate factors that are associated with oncogenic HPV detection in Kenyan women.

**Methods:**

Women without cervical dysplasia were enrolled in a longitudinal study. Data from enrollment are presented as a cross-sectional analysis. Demographic and behavioral data was collected, and HPV typing was performed on cervical swabs. HIV-uninfected women (*n* = 105) and HIV-infected women (*n* = 115) were compared for demographic and behavioral characteristics using t-tests, Chi-square tests, Wilcoxon sum rank tests or Fisher’s exact tests, and for HPV detection using logistic regression or negative binomial models adjusted for demographic and behavioral characteristics using SAS 9.4 software.

**Results:**

Compared to HIV-uninfected women, HIV-infected women were older, had more lifetime sexual partners, were less likely to be married, were more likely to regularly use condoms, and were more likely to have detection of HPV 16, other oncogenic HPV types, and multiple oncogenic types. In addition to HIV, more lifetime sexual partners was associated with a higher number of oncogenic HPV types (aIRR 1.007, 95% CI 1.007–1.012). Greater travel distance to the clinic was associated with increased HPV detection (aOR for detection of ≥ 2 HPV types: 3.212, 95% CI 1.206–8.552). Older age (aOR for HPV 16 detection: 0.871, 95% CI 0.764–0.993) and more lifetime pregnancies (aOR for detection of oncogenic HPV types: 0.706, 95% CI, 0.565–0.883) were associated with reduced detection.

**Conclusion:**

HIV infection, more lifetime sexual partners, and greater distance to health-care were associated with a higher risk of oncogenic HPV detection, in spite of ART use in those who were HIV-infected. Counseling of women about sexual practices, improved access to health-care facilities, and vaccination against HPV are all potentially important in reducing oncogenic HPV infections.

## Background

Cervical cancer is one of the most common malignancies in women living in sub-Saharan African countries, including Kenya [1]. The incidence (15 per 100,000 women per year) and mortality rate (12 per 100,000 women per year) of cervical cancer in Kenya far exceed the rates for women living in the United States (4 and 1 per 100,000 women per year, respectively) [2]. Oncogenic types of human papillomavirus (“high-risk”, or HR-HPV) are the causative agents of cervical cancer [3–5]. However, the reasons why some, but not all, women develop malignant consequences of HR-HPV infection are poorly understood.

Women who are infected with the human immunodeficiency virus (HIV) have a higher prevalence of HR-HPV infection compared to HIV-uninfected women [6–9]. HIV infection is common in Kenya: among Kenyan women aged 15–64 years, the prevalence of HIV was 6.9% in 2012, the last year that careful surveys were performed [10].

For women living with HIV, it is critical that the epidemiology of HPV infections and the effects of ART on HPV infection are better understood, because the incidence of cervical cancer has failed to decline in the same manner that other HIV-associated cancers have declined in the era of ART [11, 12]. We need to better understand how ART use alters oncogenic HPV persistence, multiple oncogenic HPV co-infections, and the effects of lesser-studied HPV types in HIV-infected women living in sub-Saharan Africa. In addition, while immunosuppression caused by HIV accounts for much of the high incidence of cervical cancer in Kenya, additional co-factors are likely to play a role, as cervical cancer is also very common in HIV-uninfected Kenyan women.

A study was therefore conducted to identify potentially modifiable behavioral and biological factors associated with detection of oncogenic HPV in HIV-infected and HIV-uninfected women from western Kenya. This report describes a cross-sectional analysis of HPV distribution patterns among these Kenyan women at enrollment into the longitudinal study, as well as demographic and behavioral factors that potentially influence the risk of HPV detection.

## Methods

### Objectives of the overall project

The data reported here is a cross-sectional analysis of women at the beginning of a longitudinal study whose aim was to recruit a balanced (HIV-infected and HIV-uninfected) cohort of 220 women without evidence of cervical disease (based on visual inspective with acetic acid, or VIA). The longitudinal study was designed to examine biological, behavioral, and environmental factors that contribute to the risk detection of oncogenic HPV during 4 years of observation. The project, which is being conducted through the AMPATH-Oncology Institute located in Eldoret, Kenya includes the study described here and a parallel study of optimal treatments for cervical dysplasia in Kenyan women who are HIV-infected.

### Enrollment of participants

Women were enrolled from September 2015 to October 2016 at the AMPATH Cervical Cancer Screening Program (CCSP) at MTRH in Eldoret. Women aged 18–45 years living within 30 km of Eldoret presenting for screening at the CCSP were asked to participate if they had a normal VIA that day and were willing to return quarterly for 4 years. The study and consent information were reviewed and participants received a written copy of the consent (English or Swahili). Exclusion criteria included history of an abnormal VIA or Pap smear, diagnosis of cervical intraepithelial lesion (CIN) or cervical cancer, clinical signs or symptoms of *C. trachomatis* (CT) or *N. gonorrhoeae* (GC), current pregnancy, inability to consent, or medical illness that rendered the patient unable to attend visits. Participants identified several ways to be located, and those without a cell phone were provided loaned phones. Participants were provided phone credit (50 Ksh per month) to contact study nurses if needed. All women were compensated for their time and effort at each visit (1000 Ksh).

Structured face-to-face interviews of enrollees by trained researchers were conducted at enrollment to capture social, behavioral, and biological information, including age, marital status, educational level, home ownership, walking distance to the local clinic, existing or prior medical conditions, number of lifetime sexual partners, age of sexual debut, percentage of condom-protected coital events, years of accumulative contraceptive usage, number of lifetime pregnancies, history of cervical cancer screening, history of tobacco use, and history of CT or GC. For HIV-infected women, variables were collected from the AMPATH Medical Record System (AMRS) at enrollment including date of HIV diagnosis, anti-retroviral therapy (ART), HIV viral load, and CD4 count.

### Sample collection

At enrollment, a nurse or physician collected two cervical swabs as part of the pelvic examination and inspection of the cervix: one for HPV testing and one for CT/GC testing. Swabs were placed in standard transport media then frozen at − 80 °C in the AMPATH Reference Laboratory. Oral rinse specimens and anal swabs were also collected and frozen at − 80 °C for future studies. Serum was collected from all participants and frozen at − 20 °C for future studies.

### HPV testing

Specimens were transported on dry ice to the Kenya Medical Research Institute-University of Massachusetts Medical School (KEMRI-UMMS) laboratory for processing and subsequent genotyping. Dry swab samples were eluted in 1 mL 1X PBS, and 250 μL aliquots of eluted samples were used for DNA extraction; the remaining eluted sample was stored at − 20 °C. DNA was extracted following the Qiagen DNA extraction protocol (QIAamp® MinElute® Media Kit 50) (Qiagen, Hilden, Germany). The Roche Linear Array was used to determine HPV types (Roche Molecular Systems, Inc., Branchburg, NJ USA) as previously described [13]. HPV 16-positive, negative, and human β-globin (used to assess specimen adequacy) controls provided by the manufacturer were tested with each batch of samples.

HPV types were grouped into “high-risk” (HR-HPV) and “low-risk” (LR-HPV) based on the designation in the Roche Linear Array instructions, or HR-HPV types as designated by the International Agency for the Research on Cancer (IARC) [14]. HPV types were further grouped into A9 and A7 types [15]. The specific HPV types included in each group are detailed in Results.

### Testing for CT/GC

Analysis of cervical swab specimens for CT/GC was performed using the *m2000rt* or *m2000sp* platform (Abbott Laboratories, Abbott Park, IL). Asymptomatic women who tested positive for GC/CT were contacted, treated, and retained in the study, as opposed to women with clinical signs or symptoms of GC/CT at enrollment who were excluded from the study.

### Statistical analysis

Demographic and behavioral characteristics of participants were summarized by descriptive statistics and compared between HIV-uninfected and HIV-infected women. Age, age of first sex, years of accumulative birth control use and number of pregnancies were normally distributed continuous variables and were compared based on t-tests; number of lifetime sex partners was a continuous variable with a right skewed distribution and was compared between groups based on Wilcoxon rank sum test; education level, home ownership, walking distance to health care, having medical conditions in the past 12 months, condom use, life history of cervical cancer screening, and life history of CT or GC were binary variables and were compared based on Chi-square tests; life history of tobacco use was a binary variable with small cell size and was compared based on Fisher’s exact test. Unadjusted univariate logistic regression or negative binomial regression models were fit to examine associations between HIV infection and HPV detections. Furthermore, adjusted logistic regression or negative binomial regression models examining associations between HIV and HPV were fit by adding demographic and behavioral variables (described above in Interview and Questionnaire) into the unadjusted models as covariates. Logistic regression models were fit for each of the binary outcomes, i.e., type-specific HPV, HPV combination, ≥2 types of any HPV, and ≥ 2 types of high-risk HPV; negative binomial models were chosen for the count outcomes, i.e., number of HPV types and number of high-risk HPV types, because the variance of each of the count outcome was greater than it’s mean. In the adjusted logistic regression or negative binomial regression models, all demographic and behavioral variables were included as covariates in the model without exclusion.

The sample size for the project (220 women, approximately half of whom are HIV-infected) was chosen to ensure sufficient power to achieve an accurate description of HPV prevalence and type distribution in HIV-uninfected and HIV-infected participants in Kenya. This sample size was predicted to provide 80% power to detect a 20% difference in detection of oncogenic HPV types (assuming 40% oncogenic HPV detection in HIV-uninfected women and 60% in HIV-infected women) by using a two-sided Chi-square test at 0.05 significance level. All analyses were performed using SAS Version 9.4 (Cary, NC).

### Ethics considerations

Study approval was granted from the local review board at Moi Teaching Referral Hospital (MTRH) and Moi University, Eldoret, Kenya, the Kenya Medical Research Institute’s Scientific and Ethics Review Unit (KEMRI-SERU) and the Institutional Review Board of Indiana University School of Medicine.

## Results

### Overall characteristics of participants

A total of 285 women were approached for participation; 223 women were enrolled. The HIV status was not available for one woman, and cervical samples from two women were inadequate based on negative β-globin control results. These three women were therefore excluded from the analysis, leaving 220 evaluable participants: 115 HIV-infected and 105 HIV-uninfected women.

Characteristics of HIV-infected and HIV-uninfected women are shown in Table 1. Compared to HIV-uninfected women, HIV-infected women were older and less likely to be married, had more lifetime sexual partners, had a lower age of first sexual intercourse, used condoms on a more regular basis during sexual intercourse, and were more likely to have had cervical cancer screening during their lifetime. Other characteristics measured were not different between HIV-infected/uninfected women.

**Table 1:** Characteristics of HIV-infected and HIV-uninfected women at enrollment into the study

Characteristic	Hiv-infected (*N* = 115)	Hiv-uninfected (*N* = 105)	*P*-value
Median age (range, IQR)	37 (21–48, 33–41)	33 (21–46, 29–38)	0.008^3^
Married (n, %)	41 (35.7)	71 (67.3)	<.001^4^
More than secondary school education (n, %)	10 (8.7)	16 (15.2)	0.132^4^
Home ownership (n, %)	19 (16.5)	28 (26.6)	0.067^4^
Walking distance to health care ≥60 min (n, %)	12 (11.4)	16 (13.6)	0.687^4^
Having medical conditions in the past 12 months^1^ (n, %)	104 (90.4)	101(96.2)	0.091^4^
Median number of lifetime sex partners (range, IQR)	4 (1–260) (3–8)	3 (1–10) (1–4)	<.001^5^
Median age of first sex (range, IQR)	17.1 (12–26, 15–19)	18.1 (8–30, 16–20)	0.025^3^
Condom use > 75% of all coital events (n, %)	65 (56.5)	13 (12.5)	<.001^4^
Median years of accumulative birth control use^2^ (range, IQR)	4 (0.1–20.3, 2–8)	4 (0.1–19, 2–7)	0.483^3^
Median number of pregnancy (range, IQR)	3 (1–9, 2–4)	3 (0–9, 2–4)	0.633^3^
Life history of cervical cancer screening (n, %)	68 (59.1)	32 (30.5)	<.001^4^
Life history of tobacco use (n, %)	6 (5.3)	1 (1.0)	0.121^6^
Life history of *Chlamydia trachomatis* or *Neisseria gonorrhea* (n, %)	10 (8.7)	6 (5.8)	0.395^4^

^1^Tuberculosis, malaria, hypertension, diabetes, pneumonia, typhoid, or stomach pain/ulcer

^2^Intrauterine device, injectable contraceptive, or oral contraceptive pills

^3^*P*-value from t-test

^4^*P*-value from Chi-square test

^5^*P*-value from Wilcoxon rank sum test

^6^*P*-value from Fisher’s exact test

### HIV-specific characteristics

Data regarding HIV diagnosis and treatment history were available for all HIV-infected participants. The median duration between HIV diagnosis and study enrollment was 7.2 years (range 0–14.2, IQR 4.1–10.3) for 114 of 115 women whose date of HIV diagnosis was available. Among all 115 HIV-infected participants, 105 (91.3%) were receiving ART at enrollment. The median years receiving ART was 4.2 years (range 0–10.5, IQR 1.6–6.4) for 104 women whose ART initiation date was available.

The median CD4 count at the time of HIV diagnosis was available for 113 of 115 HIV-infected women and was 471 cells/uL (range 0–1382, IQR 310–612). The median CD4 count for 112 of 115 women whose results were available at enrollment was 538 cells/uL (range 17–1474, IQR 377–782).

All 115 HIV-infected women had HIV viral load measured at enrollment. For 83 of 115 women (72.2%), the HIV viral load was undetectable (< 40 copies/mL in the Roche Amplicor Assay, which was assigned a value of 0 copies/mL). For all 115 HIV-infected women, the mean HIV viral load was 46,619 copies/mL (range 0–4,498,711; IQR 0–76), and the median was 0 copies/mL. For 32 of 115 HIV-infected women (27.8%) who had a detectable HIV viral load (> 40 copies/mL), the mean viral load was 167,432 copies/mL (range 43–4,498,711; IQR 217–23,830), and the median was 3842 copies/mL.

### HPV type distribution

Regardless of HIV status, one or more of the 37 HPV types in the Roche Linear Array assay were detected in 105 of all 220 participants (47.7%). Specimens from 83 of these 220 women (37.7%) were positive for any HR-HPV type included in the Roche Linear Array assay (HPV 16, 18, 26, 31, 33, 35, 39, 45, 51, 52, 53, 56, 58, 59, 66, 67, 68, 69, 70, 73, 82, IS39). One or more HR-HPV types defined by IARC (HPV types 16, 18, 31, 33, 35, 39, 45, 51, 52, 56, 58, 59, 66) were detected in 70 of 220 (31.8%) participants.

### HPV type distribution by HIV status

**Fig. 1:**
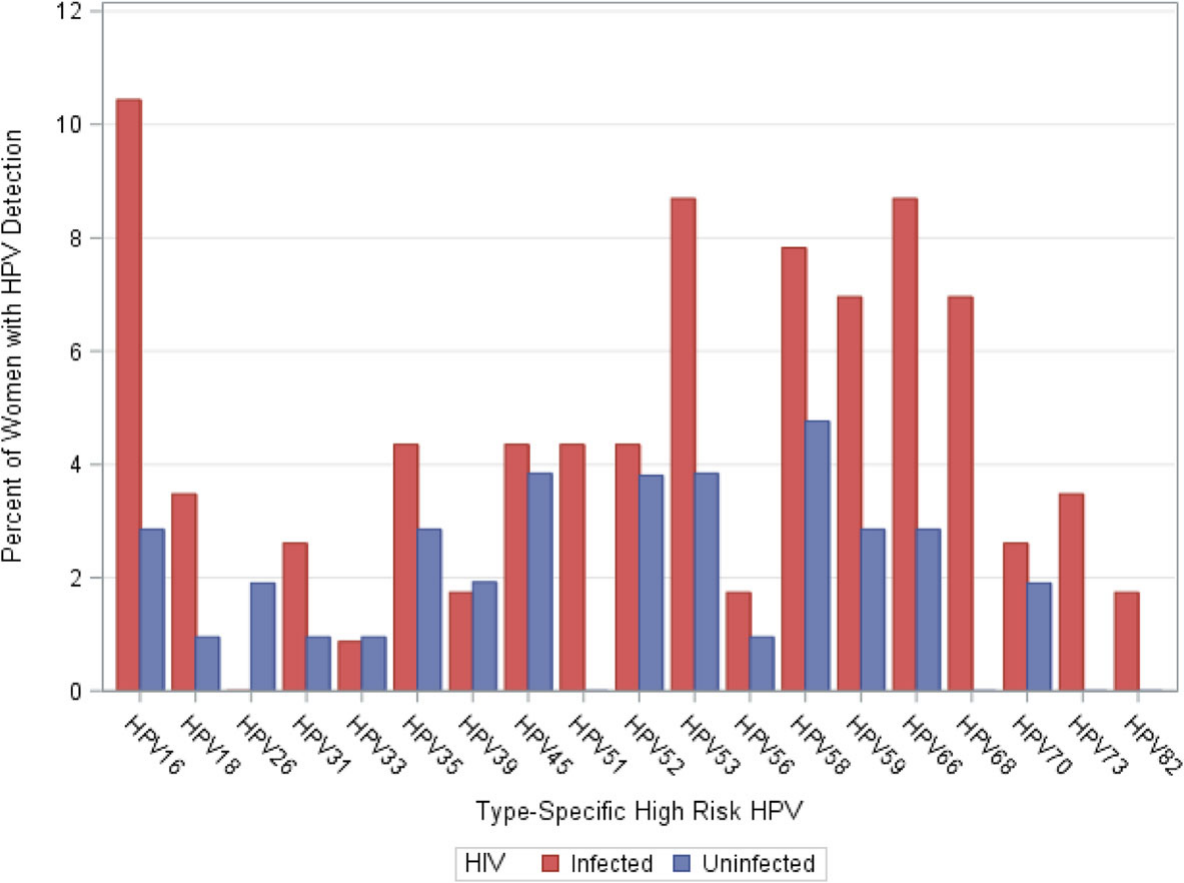
Detection of individual High Risk (HR)-HPV types in HIV-infected and HIV-uninfected women

Detection of individual HR-HPV types by HIV status is shown in Fig. 1. The four most frequently detected HR-HPV types among 115 HIV-infected women were HPV 16 (*n* = 12, 10.4%), HPV 53 (*n* = 10, 8.7%), HPV 66 (*n* = 10, 8.7%), and HPV 58 (*n* = 9, 7.8%). The four most frequently detected HR-HPV types in 105 HIV-uninfected women were HPV 58 (*n* = 5, 4.8%), HPV 45 (*n* = 4, 3.8%), and HPV 52 and 53 (both *n* = 4, 3.8%).

Results from adjusted regression models demonstrated that HPV of any type (aOR = 3.117, 95% CI = 1.460–6.654), HR-HPV types (aOR = 3.301, 95% CI = 1.483–7.344), IARC-defined HR-HPV types (aOR = 2.593, 95% CI = 1.147–5.860), A9 types (aOR = 2.569, 95% CI = 1.023–6.446), HPV 16 (aOR = 9.510, 95% CI = 1.757–51.463), A7 types (aOR = 3.813, 95% CI = 1.284–11.319), non-HPV 18 A7 types (aOR = 3.320, 95% CI = 1.037–10.629), and HPV 68 (aOR = 10.057, 95% CI = 1.803 - infinity) were all detected significantly more often in HIV-infected women compared to HIV-uninfected women (Table 2). Regarding detection of multiple HPV types, 38 of 115 (33.0%) HIV-infected women had two or more HPV types detected compared to 11 of 105 (10.5%) HIV-uninfected women (aOR = 6.111, 95% CI = 2.265–16.483), 23 of 115 (20.0%) HIV-infected women had two or more HR-HPV types detected compared to 7 of 105 (6.7%) HIV-uninfected women (aOR = 3.705, 95% CI = 1.195–11.488). The number of HPV types detected was 1.3 (Standard Deviation 1.7, Range 0–7) for HIV-infected women and 0.6 (Standard Deviation 1.1, Range 0–8) for HIV-uninfected women (aOR = 2.585, 95% CI = 1.611–4.418) (Table 2). The number of HR-HPV types detected was 0.9 (Standard Deviation 1.2, Range 0–6) for HIV-infected women and 0.4 (Standard Deviation 0.7, Range 0–4) for HIV-uninfected women (aOR = 2.519, 95% CI = 1.476–4.299) (Table 2).

**Table 2:** HPV type distribution among HIV-infected and HIV-uninfected women at enrollment

HPV TYPES	HIV-INFECTED (*N* = 115) n (%) or mean (SD, range)	HIV-UNINFECTED (*N* = 105) n (%) or mean (SD, range)	UNADJUSTED MODEL		ADJUSTED MODEL^i^	
			OR or IRR^l^ (95% CI)	*P*-value	aOR or aIRR^m^ (95% CI)	*P*-value
Any HPV type^a^	68 (59.1)	37 (35.2)	2.659 (1.540–4.592)	<.001	3.117 (1.460–6.654)	0.003
HR-HPV^b^	54 (47.0)	29 (27.6)	2.319 (1.321–4.073)	0.003	3.301 (1.483–7.344)	0.003
IARC HR-HPV^c^	45 (39.1)	25 (23.8)	2.057 (1.146–3.691)	0.016	2.593 (1.147–5.860)	0.022
A9 HPV^d^	27 (23.5)	16 (15.2)	1.706 (0.860–3.385)	0.126	2.569 (1.023–6.447)	0.045
HPV 16	12 (10.4)	3 (2.9)	3.961 (1.086–14.454)	0.037	9.510 (1.757–51.463)	0.009
Non-HPV 16 A9^e^	20 (17.4)	13 (12.4)	1.490 (0.700–3.169)	0.301	1.590 (0.583–4.333)	0.365
A7 HPV^f^	23 (20.0)	9 (8.6)	2.667 (1.172–6.066)	0.019	3.813 (1.284–11.319)	0.016
HPV 18	4 (3.5)	1 (1.0)	3.748 (0.412–34.080)	0.241	4.583 (0.311–67.632)	0.268
HPV 68^j^	8 (7.0)	0 (0)	10.684 (2.684 - inf^k^)	0.010	10.057 (1.803 - inf^k^)	0.022
Non-HPV 18 A7^g^	20 (17.4)	8 (7.6)	2.553 (1.072–6.077)	0.034	3.320 (1.037–10.629)	0.043
Any LR-HPV^h^	37 (32.2)	18 (17.1)	2.293 (1.208–4.352)	0.011	2.122 (0.913–4.932)	0.080
≥2 Types Any HPV	38 (33.0)	11 (10.5)	4.217 (2.021–8.799)	<.001	6.111 (2.265–16.483)	<.001
≥2 Types HR-HPV	23 (20.0)	7 (6.7)	3.500 (1.433–8.544)	0.006	3.705 (1.195–11.488)	0.023
Number of HPV types	1.3 (1.7, 0–7)	0.6 (1.1, 0–8)	2.320 (1.557–3.457)	<.001	2.585 (1.611–4.148)	<.001
Number of HR-HPV types	0.9 (1.2, 0–6)	0.4 (0.7, 0–4)	2.294 (1.463–3.597)	<.001	2.519 (1.476–4.299)	0.001

^a^HPV 6, 11, 16, 18, 26, 31, 33, 35, 39, 40, 42, 45, 51, 52, 53, 54, 55, 56, 58, 59, 61, 62, 66, 67, 68, 69, 70, 71, 72, 73, 81, 82, 83, 84, CP6108, IS39

^b^High-Risk HPV 16, 18, 26, 31, 33, 35, 39, 45, 51, 52, 53, 56, 58, 59, 66, 67, 68, 69, 70, 73, 82, IS39

^c^HPV 16, 18, 31, 33, 35, 39, 45, 51 52, 56, 58, 59, 66

^d^HPV 16, 31, 33, 35, 52, 58

^e^HPV 31, 33, 35, 52, 58

^f^HPV 18, 39, 45, 59, 68

^g^HPV 39, 45, 59, 68

^h^Low-Risk HPV 6, 11, 40, 42, 54, 55, 61, 62, 64, 71, 72, 81, 83, 84, CP6108

^i^Age, marital status, educational level, home ownership, distance to health care, having medical conditions in the past 12 months, life-time sexual partners, age of first sex, condom use, accumulative years of birth control use, total number of pregnancy, and history of cervical cancer screening, smoking history, history of CT or GC were adjusted as covariates in the logistic regression model or the negative binomial regression model

^j^Exact logistic regression model was conducted for HPV 68 due to low cell sizes

^k^Infinity

^l^Odds ratio or Incidence rate ratio; ^m^Adjusted odds ratio or adjusted incidence rate ratio

### Demographic and behavioral characteristics related to HPV detection

In addition to HIV status, demographic and behavioral characteristics significantly associated with HPV detection were identified from regression models (Table 3). Specifically, older age was associated with a lower rate of HPV 16 detection (adjusted Odds Ratio (aOR) 0.871, 95% CI 0.764–0.993), but not other HR-HPV. Living at a walking distance of 60 min or more from the clinic was associated with an increased likelihood of detection of two or more HPV types (aOR 3.212, 95% CI 1.206–8.552). A greater number of lifetime sexual partners was associated with detection of a higher number of overall HPV types (Incidence Rate Ratio 1.007, 95% CI 1.001–1.012) and a higher number of HR-HPV types (aOR 1.007, 95% CI 1.001–1.013). A higher number of life time pregnancies was associated with reduced detection of HR-HPV (aOR 0.706, 95% CI 0.565–0.883), IARC HR-HPV types (aOR 0.624, 95% CI 0.486–0.801), non-HPV 16 A9 types (aOR 0.714, 95% CI 0.523–0.975), and A7 HPV types (aOR 0.733, 95% CI 0.583–0.998). Other characteristics in the models (educational level, home ownership, existing or prior medical conditions, age of first sex, percentage of condom-protected coital events, years of accumulative contraceptive usage, history of cervical cancer screening, history of tobacco use, and history of CT or GC) were not associated with increased or decreased HPV detection (data not shown).

**Table 3:** Demographic and behavioral characteristics at enrollment that were significantly associated with HPV detection identified from regression models

Demographical and behavioral characteristics	HPV	aOR/aIRR^a^ (95% CI)	*P*-value
Age	HPV 16	0.871 (0.764–0.993)	0.039
Walking distance > 60 min to clinic	≥2 HPV types	3.212 (1.206–8.552)	0.020
Number of lifetime sexual partners	Number of HPV types	1.007 (1.001–1.012)	0.026
	Number of HR-HPV types	1.007 (1.001–1.013)	0.018
Number of lifetime pregnancies	HR-HPV types	0.706 (0.565–0.883)	0.002
	IARC HR-HPV types	0.624 (0.486–0.801)	<.001
	Non-HPV 16 A9 types	0.714 (0.523–0.975)	0.034
	A7 HPV types	0.733 (0.538–0.998)	0.049

^a^Adjusted odds ratio or adjusted Incidence rate ratio

## Discussion

Cervical cancer occurs more often in women infected with HIV than in HIV-uninfected women, and causes death in more women living in sub-Saharan Africa than any other cancer [16–19]. While HIV is a critical factor in accelerating the natural history of cervical cancer, it is likely that additional factors also play a role. Approximately half of the women in were HIV-infected.

The data presented here represent the enrollment analysis of 220 women. Compared to HIV-uninfected women, HIV-infected women were older and less likely to be married, had more lifetime sexual partners, had a lower age of first sexual intercourse, used condoms on a more regular basis, and were more likely to have had cervical cancer screening during their lifetime. HPV was frequently detected in both groups of women. As expected, all types of HPV and HR-HPV types were detected more often in HIV-infected women than in women without HIV. HR-HPV types as defined by IARC were also frequently detected: 39.1% of HIV-infected women compared to 23.8% of HIV-uninfected women. Multiple HPV types and multiple HR-HPV types were also detected in a higher percentage of HIV-infected women than in HIV-uninfected women.

For individual oncogenic HPV types, HPV 16, the type responsible for the greatest percentage of cervical cancers worldwide was detected more often in HIV-infected women than in those without HIV. Other A9 HPV types were not detected differently at a significant level between HIV groups. For A7 HPV types, only HPV 68, an A7 HR-HPV type included in the Linear Array assay but not included in the IARC group of oncogenic types, was detected more often in HIV-infected women than in HIV-uninfected women. The importance of HPV 68 is not yet known, but will be further evaluated.

Several prior cross-sectional studies have examined HPV type distribution in African women. HPV detection and the specific types identified varied in these studies depending on the country where the study was performed and the sampling/typing methods used [20–22]. These studies consistently indicate that HIV-infected women have a higher rate of HPV infection compared to HIV-uninfected women [23–29]. However, few longitudinal studies of African women have been conducted in HIV-infected African women, so data on HPV persistence, episodic detection, and clearance are not available.

In a cross-sectional study of 498 HIV-infected Kenyan women, HPV was detected in 68.7%; 52.6% had an HR-HPV infections, and 40.2% were infected with multiple HPV types [28]. Compared to HIV-infected women not receiving ART, those receiving ART for 2 years or longer, especially those with CD4 counts greater than 500 cells per uL, had less detection of HR-HPV compared to women not receiving ART.

Three other studies of HIV-infected sub-Saharan women also indicate a positive impact of ART on reducing HR-HPV detection, as reviewed by Menon et al. [30]. A meta-analysis also concluded that ART use, after adjusting for duration of use and CD4 count, was associated with reduced rate of HR-HPV in HIV-infected women [31]. In contrast, a study of HIV-infected Ugandan women, nearly all of whom had HR-HPV detected prior to ART initiation, had no reduction of HPV detection during a follow-up period limited to 6 months [32].

Most HIV-infected women in our study were receiving ART at enrollment and had documented suppression of HIV replication. Because only a small number of HIV-infected women were not receiving ART, it was not possible to determine the effect of ART on HPV detection.

Behavioral, socio-economic and environmental factors for women living in sub-Saharan Africa are likely to influence HPV infection rates and development of HPV-associated malignancies. We attempted to determine factors in addition to HIV infection that influenced detection of HPV. Factors in the regression analysis that increased the risk of HPV detection (in addition to HIV infection) included a greater distance of travel to the clinic and a higher number of lifetime sexual partners. Factors in the regression analysis that decreased the risk of HPV detection included older age and a higher number of lifetime pregnancies.

Our study is limited by the modest number of women enrolled. This study is also limited by the lack of serological data, which could potentially identify women who have been infected in the past with specific HPV types but have a negative PCR assay, due to a HPV infection that is below the limit of detection [33].

Counseling of girls and young women and improved access to clinics and other health-care facilities may be important in reducing oncogenic HPV infections. Because vaccination against HPV is effective in preventing infection with oncogenic HPV types, it is important that all women are vaccinated, whether they are HIV-infected or HIV-uninfected [34, 35].

## Conclusions

Oncogenic types of HPV were detected in a high percentage of all women, but were more frequently detected in HIV-infected women than in HIV-uninfected Kenyan women. Multiple HR-HPV types were also detected in a higher percentage of HIV-infected women than in HIV-uninfected women. For individual oncogenic HPV types, HPV 16 and HPV 68 were detected more often in HIV-infected women than in those without HIV. In addition to HIV, younger age, living at a walking distance of 60 min or more from the clinic, a greater number of lifetime sexual partners, and a smaller number of lifetime pregnancies were all associated with increased frequency of detection of oncogenic HPV types. Information from this study will be valuable in informing HPV vaccine programs in Kenya, and will guide cervical cancer screening programs in Kenya that utilize HPV DNA testing.

## Abbreviations

AMPATH: Academioc Model Providing Access to Healthcare
AMRS: AMPATH Medical Record System
ART: Anti-retroviral therapy
CCSP: AMPATH Cervical Cancer Screening Program
CIN: Cervical intraepithelial neoplasia
CT: *C.trachomatis*
GC: *N.gonorrhoeae*
HR-HPV: high-risk HPV
IARC: International Agency for the Research on Cancer
IQR: Inter-quartile range
KEMRI-SERU: Kenya Medical Research Institute’s Scientific and Ethics Review Unit
KEMRI-UMMS: Kenya Medical Research Institute-University of Massachusetts Medical School
LR-HPV: Low-risk HPV
MTRH: Moi Teaching Referral Hospital
VIA: Visual inspective with acetic acid
